# Development and Functional Analysis of Novel Genetic Promoters Using DNA Shuffling, Hybridization and a Combination Thereof

**DOI:** 10.1371/journal.pone.0031931

**Published:** 2012-03-14

**Authors:** Rajiv Ranjan, Sunita Patro, Bhubaneswar Pradhan, Alok Kumar, Indu B. Maiti, Nrisingha Dey

**Affiliations:** 1 Department of Gene Function and Regulation, Institute of Life Sciences, Department of Biotechnology, Government of India, Chandrasekherpur, Bhubaneswar, Odisha, India; 2 Kentucky Tobacco Research and Development Center (KTRDC), University of Kentucky, Lexington, Kentucky, United States of America; Nanjing Agricultural University, China

## Abstract

**Background:**

Development of novel synthetic promoters with enhanced regulatory activity is of great value for a diverse range of plant biotechnology applications.

**Methodology:**

Using the *Figwort mosaic virus* full-length transcript promoter (F) and the sub-genomic transcript promoter (FS) sequences, we generated two single shuffled promoter libraries (LssF and LssFS), two multiple shuffled promoter libraries (LmsFS-F and LmsF-FS), two hybrid promoters (FuasFScp and FSuasFcp) and two hybrid-shuffled promoter libraries (LhsFuasFScp and LhsFSuasFcp). Transient expression activities of approximately 50 shuffled promoter clones from each of these libraries were assayed in tobacco (*Nicotiana tabacum* cv. Xanthi) protoplasts. It was observed that most of the shuffled promoters showed reduced activity compared to the two parent promoters (F and FS) and the CaMV35S promoter. *In silico* studies (computer simulated analyses) revealed that the reduced promoter activities of the shuffled promoters could be due to their higher helical stability. On the contrary, the hybrid promoters FuasFScp and FSuasFcp showed enhanced activities compared to F, FS and CaMV 35S in both transient and transgenic *Nicotiana tabacum* and *Arabidopsis* plants. Northern-blot and qRT-PCR data revealed a positive correlation between transcription and enzymatic activity in transgenic tobacco plants expressing hybrid promoters. Histochemical/X-gluc staining of whole transgenic seedlings/tissue-sections and fluorescence images of ImaGene Green™ treated roots and stems expressing the *GUS* reporter gene under the control of the FuasFScp and FSuasFcp promoters also support the above findings. Furthermore, protein extracts made from protoplasts expressing the human defensin (HNP-1) gene driven by hybrid promoters showed enhanced antibacterial activity compared to the CaMV35S promoter.

**Significance/Conclusion:**

Both shuffled and hybrid promoters developed in the present study can be used as molecular tools to study the regulation of ectopic gene expression in plants.

## Introduction

In the eukaryotic cell, expression of a transgene depends upon the presence of the primary regulatory element, the promoter, which plays a major role in determining the relative level of transcription and, ultimately, gene expression and function. The promoter region of a gene expression cassette is modular, consisting of several small DNA sequence motifs (*cis-*elements). It is the combinatorial interaction of these *cis*-elements with various nuclear protein factors (*trans*-factors) that define a given promoter's strength and tissue specificity [1]. By manipulating the architecture of the promoter sequence through ‘*cis* re-arrangement’, the relative strength and tissue specificity of a promoter can be optimized, allowing the development of improved gene expression vectors. There is currently a paucity of engineered promoters designed for plant biotechnology applications, and novel approaches in the design of such promoters need to be explored extensively [2].

A number of hybrid or chimeric recombinant plant promoters have been developed recently by (a) cis-domain swapping of one promoter with the functionally equivalent domain from other heterologous promoters [3], and (b) ligating the upstream activation sequence (UAS) from one promoter to the TATA box-containing domain of another promoter [4]–[8]. The promoter designated as ‘superpromoter’ was constructed by fusing three repeats of the octopine synthase transcriptional activating element with the *mannopine synthase2*′ (*mas2*′) transcriptional activating element plus the minimal promoter region. Recently, a useful plant transformation vector has been constructed that incorporates the superpromoter [9]. Synthetic cis-element sequences in conjunction with heterologous promoters have also been used to design various plant promoters [10], [11]. The basic rationale behind developing such modified promoters lies in the notion that the transfer of the upstream DNA sequence/*cis*-element that binds a specific *trans*-factor from one promoter into a different promoter containing the TATA sequence might result in a novel regulatory or transcription model [12]. Apart from these approaches, linker scanning mutagenesis [13] and error prone PCR [14] have also been used to introduce either random or specific mutations into a promoter sequence – the objective being to alter either the orientation/arrangement of the existing *cis* elements or to insert or destroy *cis*-elements that modify the existing function of a promoter. Recently, molecular evolution (DNA shuffling), a powerful tool for introducing random mutations into DNA sequences, has been successfully used to modify DNA sequences from one or more genes for improvement of enzyme catalytic properties and stability as well as expanding the substrate specificity of number of genes [15]–[21]. Although DNA shuffling has the real potential to generate promoter libraries consisting of functional promoters of varying strength, (both constitutive and tissue specific), it has not been sufficiently exploited in promoter modification.

In the present study, we were interested in developing efficient promoters by adopting a combination of hybridization and DNA shuffling techniques. As starting genetic material, we used the *Figwort mosaic virus* full-length transcript promoter (F, −249 to +64) [22] and the *Figwort mosaic virus* sub-genomic transcript promoter (FS, −270 to +31) [23]. We have generated hundreds of modified promoters using DNA shuffling approaches in two possible combinations; single-shuffling and multiple-shuffling. We also developed a pair of hybrid promoters, viz., FuasFScp and FSuasFcp by intermolecular exchange of the important domains of F and FS promoters. Hundreds of shuffled promoter clones were obtained by one round shuffling of these two hybrid promoters individually. Activities of 300 shuffled promoter clones along with two hybrid promoters were assayed transiently in tobacco (*Nicotinia tabacum* cv. Xanthi) protoplast suspension cultures, and their activities were compared to those obtained from the parent promoters (F, FS) and the CaMV35S promoter. Furthermore, the expression analyses of hybrid promoters were carried out in transgenic tobacco and *Arabidopsis* plants. Correlation between the GUS activity and the *uidA*-mRNA levels driven by the two hybrid promoters (FuasFScp and FSuasFcp) in transgenic tobacco plants were verified. The cell-specific expression of these hybrid promoters were evaluated using ImaGene Green™ (Molecular Probe)-treated transgenic tissue employing confocal laser scanning microscopy (CLSM). The localization of GUS activities was studied using X-gluc staining of whole seedlings and tissue sections. The antibacterial activities of hybrid promoter-driven human defensin (HNP-1) protein expressed in tobacco protoplasts were compared to that obtained from the CaMV35S promoter. Hybrid promoters showed stronger activities compared to CaMV35S promoter.

In plant molecular pharming, there is a constant need for both strong/weak constitutive/tissue specific promoters with diverse sequences. The shuffled promoter libraries developed in the present study contain promoters with varying activities. These promoters along with the hybrid promoters FuasFScp and FSuasFcp with enhanced activity could be used for plant biotechnology applications.

## Materials and Methods

Restriction and modifying enzymes were purchased from Promega (Madison, WI, USA), and were used according to the manufacturer's instructions. Nytran membrane was obtained from Schleicher & Schuell (Keene, NH, USA). General chemicals, including MUG, X-gal, X-gluc, and DEPC were purchased from Sigma-Aldrich (St. Louis, USA). Platinum high fidelity *Taq* DNA polymerase and ImaGene Green™ C_12_ FDGlcU GUS Gene Expression Kit were purchased from Invitrogen (California, USA). Human α-defensin-1 (HNP-1) cloned in baculovirus expression vector was kindly provided by Prof T. Ganz, UCLA Department of Medicine USA. *Staphylococcus aureus* was obtained from IMTECH Chandigarh, India, and *E. coli* K12 (TB1) was procured from New England Biolabs, USA.

### Construction of hybrid promoters: FuasFScp and FSuasFcp

**Figure 1:**
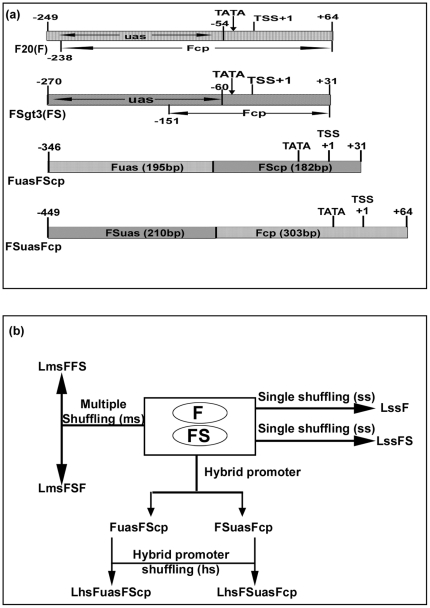
A schematic map of the parent promoters (F and FS), hybrid promoters (FuasFScp and FSuasFcp) and DNA shuffling strategy. (a) At the top, the coordinates of the respective promoters *Figwort mosaic virus* (FMV) full-length transcript promoter (F, −249 to +64), FMV sub-genomic transcript promoter (FS, −270 to +31), and two hybrid promoters (FuasFScp, −343 to +31; and FSuasFcp, −449 to +64), the relative position of the TATA box, transcription start site (TSS, +1), upsteam activation sequence (uas) and core-promoter (cp) regions marked with arrow were shown. (b) A schematic presentation of creating promoter libraries by DNA shuffling of single (F or FS), multiple (F and FS) and hybrid promoters (FuasFScp, FSuasFcp) was presented. The construction strategies of generating hybrid promoters (FuasFScp and FSuasFcp), the single shuffled libraries (LssF and LssFS), multiple shuffled libraries (LmsFFS and LmsFSF), and hybrid promoter shuffled libraries (LhsFuasFScp and LhsFSuasFcp) were described in “Materials and Methods” section.

A schematic map of parent promoters (F and FS) and hybrid promoters (FuasFScp and FSuasFcp was shown in Figure 1(a). The 195 bp long Fuas (−249 to −54, upstream activation sequence of F promoter), 314 bp long Fcp (−238 to +64, TATA box containing core-promoter sequence of F promoter) [22]; 210 bp long FSuas (−270 to −60, upstream activation sequence of FS promoter) and 182 bp long FScp (−151 to +31, TATA box containing core-promoter sequence of FS promoter) [23] were PCR amplified using promoter specific primer pairs (Table 1) having appropriate sequence to generate *Eco*RI and *Hinc*II sites at the 5′ end and *Sma*I and *Hind*III sites at the 3′ end. PCR amplifications of these promoter fragments were carried out as per protocol described earlier [24]. PCR-amplified fragments were restricted with *Eco*RI and *Hind*III, gel-purified and cloned into the corresponding sites of pBS (K+). The resulting plasmids were designated as pBSFuas, pBSFcp, pBSFSuas and pBSFScp respectively. The integrity of DNA sequences of these clones was verified by DNA sequencing as described earlier [5].

**Table 1:** Sequences of synthetic oligonucleotides.

Constructs	Forward primers (5′-3′ orientation)
F-F	CCCGTCGACAGCTGGCTTGTGGGGACCA
FS-F	CCCGTCGACTTTACAGTAAGAACTGATAACA
F-EF	CCCGAATTCGTCGACAGCTGGCTTGTGGGGACCA
FS-EF	ACTGAATTCGTCGACTCGAACATCTTGAAGGTGTAC
Fuas	CCCGAATTCGTCGACAGCTGGCTTGTGGGGACCA
FSuas	CCCGAATTCGTCGACTTTACAGTAAGAACTGATAACA
Fcp	CCCGAATTCGTCGACCGCAGTGACGACCACTTTTC
FScp	ACTGAATTCGTCGACTCGAACATCTTGA AGGTGTAC
*GFP*	ACTCTCGAGATGAGTAAAGGAGAAGAACTT
*GUS*	GATCGCGAAAACTGTGGAAT
*β-Actin*	ATGACTCAGATCATGTTTGAG
*GAPDH*	CAGTAAACGACCCGTAAATG
*HNP-1*	GAGCTCGTGACCCCAGCCATGAGG

	Reverse primers (5′-3′ orientation)
F-R	GGGCCCGGGGTGCTTAGCCTCTCATTGAAG
FS-R	GGGCCCGGGGGTCTTAGCCTCTCATTGAAG
F-HR	GGGAAGCTTCCCGGGGTGCTTAGCCTCTCATTGAAG
FS-HR	ACTAAGCTTCCCGGGCACTCCCCCTCT CTAAAAATT
Fuas	GGGAAGCTTCCCGGGTTTTGTGGTCGTCACTGCG
FSuas	GGGAAGCTTCCCGGGAAATCATACGTCAGCGCTTA
Fcp	GGGAAGCTTCCCGGGGGTCTTAGCCTCTCATTGAAG
FScp	ACTAAGCTTCCCGGGCACTCCCCCTCT CTAAAAATT
*GFP*	ACTGAGCTCTTATTTGTATAGTTCATCCATG
*GUS*	TAATGAGTGACCGCATCGAA
*β-Actin*	AGCCTTCGCAATCCACATCTG
*GAPDH*	GCCAGTTGGTGTTAATGTTT
*HNP-1*	CTCGAGCAAGCTCAGCAGC

The Fcp promoter fragment was isolated from pBSFcp as *Hinc*II-*Hind*III fragment; and inserted into the *Sma*I and *Hind*III sites of pBSFSuas to generate pBSFSuasFcp clone. Similarly the FScp fragment was inserted into the pBSFuas to generate pBSFuasFScp clone. All plasmid inserts were subjected to nucleotide sequencing and the upstream activating sequence (uas) portion was shown to be linked to the TATA-containing promoter) in both hybrid promoters.

All native and modified promoter fragments, viz., Fuas, FSuas, Fcp, FScp, FuasFScp and FSuasFcp were isolated by *Eco*RI and *Hind*III restriction digestions from corresponding pBSK (+) based clones and sub-cloned into corresponding sites of plant protoplasts expression vector pUCPMAGUS by replacing the CaMV35S promoter as described earlier [24]. The resulting clones were designated as pUPFuasGUS, pUPFSuasGUS, pUPFcpGUS, pUPFScpGUS pUPFuasFScpGUS and pUPFSuasFcpGUS, respectively.

### Construction of promoter-libraries by DNA shuffling of single, multiple and hybrid promoters

#### DNA shuffling of single promoter

A schematic flowchart illustrating the construction of six shuffled promoter libraries was shown in Figure 1(b). The F and FS promoter fragments (Figure 1a) were PCR-amplified from respective plasmid clones pFMV20 containing F20 (F) promoter [22] and pFS3 containing FSgt3 (FS) promoter [23] using promoter specific primer pairs (Table 1) as described earlier [24]. An aliquot of 5.0 µg of PCR product (promoter DNA) was digested with 0.5 U of *DNaseI* (Promega, USA) at room temperature for 10 min. After heat inactivation at 65°C digested products were gel purified. The fragment assembly PCRs (self-primed PCRs) was carried out using 4 µl of the respective digested products under following PCR condition: denaturation (94°C for 30 sec), annealing (42°C for 30 sec) and extension (72°C for 30 sec) for 25 cycles. Rescue-PCRs (33 cycles) were performed under following conditions: denaturation (94°C for 30 sec), annealing (58°C for 30 sec), extensions (72°C for 2 min) in presence of 1 µl of the assembled PCR product using Taq DNA polymerase with appropriately designed primers (Table 1) to generate *Eco*RI at 5′-end and *Hind*III at 3′-end. Rescue PCR products of F and FS promoters were subjected to restriction digestion by *Eco*RI and *Hind*III and cloned into the corresponding sites of the plant protoplast expression vector pUCPMAGUS [24] as described earlier to generate two promoter- libraries LssF and LssFS from single promoter F and FS, respectively.

#### DNA shuffling of multiple promoters

An aliquot of 5.0 µg (1∶1, wt/wt) mixture of F and FS promoter DNA was subjected to *DNaseI* digestion and subsequently fragment assembly PCR (self-primed) was carried out as described above. Two rescue PCRs (R-PCR-FFS and R-PCR-FSF) were carried out as described earlier using appropriately designed primers (Table 1) The PCR amplified products of both rescue PCRs (R-PCR-FFS and R-PCR-FSF) were cloned into pUCPMAGUS vector to generate two multiple shuffled promoter libraries LmsFFS and LmsFSF.

#### DNA shuffling of hybrid promoters

An aliquot of 5 µg of DNA (1∶1) wt/wt of hybrid promoters FuasFSCP and FSuasFCP individually was digested by *DNaseI* and assembly PCR were carried out as described earlier. Two rescue PCRs (R-PCR-FuasFScp and R-PCR-FSuasFcp) were carried out as described earlier using appropriately designed primers. (Table 1) Activities of about 50 positive clones from each library confirmed by *Eco*RI and *Hind*III digestions coupled to *GUS* reporter.

### Protoplast isolation, electroporation and Transient assay of shuffled promoters

Isolation and electroporation of protoplasts from tobacco cell suspension culture (*Nicotiana tabacum* cv. Xanthi Brad) were performed following published procedures as previously described [24]. Transient GUS activities of 50 shuffled promoter clones from each of the following promoter libraries LssF (ssF-1 to ssF-50), LssFS (ssFS-1 to ssFS-50), LmsFFS (ms FFS-1 to msFFS-50), LmsFSF (msFSF-1 to msFSF-50), LhsFuasFScp (hsFuasFScp-1 to hsFuasFScp-50) and LhsFSuasFcp (hsFSuasFcp-1 to hsFSuasFcp-50) along with F, FS and CaMV35S promoter constructs were carried out as described earlier [24]. GUS activities in transformed protoplasts were measured after 20 hrs of incubation at 28°C as described earlier [25], and protein was estimated according to the method of Bradford [26] using BSA as a standard. The average activities of these promoter constructs were expressed as the mean of three successive independent experiments.

### *In silico* studies of native and hybrid promoter sequences

*In Silico* based multiple sequence alignments were performed for few selected promoters showing less promoter activity using ClustalW2 (www.ebi.ac.uk/Tools/nsa/clustalw90) [27] to identify the position of different random mutations or deletions in shuffled promoter sequence. The sequence of FuasFScp (hybrid) promoter was aligned with two hybrid shuffled promoter clones (LhsFuasFScp-1 and LhsFuasFScp-18) from the LhsFuasFcp shuffled library those showed much decreased promoter activity than the FuasFScp promoter. Similarly, the sequence of FSuasFcp promoter was compared with sequence of LhsFSuasFcp-12 and LhsFSuasFcp-28 promoter clones from LhsFSuasFcp library those showed much decreased promoter activity than the FSuasFcp promoter by ClustalW2 [27].

Free energy profile (helical stability) of the above mentioned shuffled promoters and their wild type promoters (FuasFScp and FSuasFcp) sequences were also obtained using the software web-thermodyn (http://www.gsa.buffalo.edu/dna/dk/WEBTHERMODYN/) with step size 1 and window size 10 keeping the default parameters as follows: temperature, 37°C and salt concentration,10 mM [28].

### Construction of promoter-GFP expression vectors for transient expression assay using CLSM

The *GFP* cDNA gene was PCR-amplified using synthetic primer pair (Table 1) to generate a fragment of general structure 5′-*Xho*I- GFP-*Sst*I-3′ that was inserted into the corresponding sites of pUPFuasGUS, pUPFSuasGUS, pUPFcpGUS, pUPFScpGUS, pUPFGUS, pUPFSGUS, pUPFuasFScpGUS and pUPFSuasFcpGUS replacing the *GUS* gene to generate following plasmids pUPFuasGFP, pUPFSuasGFP, pUPFcpGFP, pUPFScpGFP, pUPFGFP, pUPFSGFP pUPFuasFScpGFP and pUPFSuasFcpGFP, respectively.

Protoplast electroporated with *GFP* constructs were excited at 488 nm and the fluorescence emissions were collected between 501 and 598 nm as described earlier [29]. Following image acquisition, GFP fluorescence intensities were quantified using the LAS AF Software attached to the confocal system as per the instructions of Leica Microsystems. The GFP fluorescence intensities from approximately 100 individual protoplasts were assayed, and the mean data were presented with respective ± SD.

### Transient agro-infiltration assay of shuffled promoters in tobacco

Twenty six shuffled promoter clones from six shuffled promoter libraries showing higher transient expression activity than the CaMV35S promoter were further evaluated in transient agro-infiltration assay *in-vivo* using whole tobacco plants. These promoters were isolated as *Eco*RI and *Hind*III fragments from respective pUCPMAGUS vector based corresponding clones and inserted into the corresponding sites of the pKYLXGUS vector replacing the CaMV35S promoter as described earlier [24]. *Agrobacterium tumefaciens* strain C58C1:pGV3850 was transformed with these twenty six shuffled promoter clones (pKYLXGUS based) individually following the published freeze thaw method as described earlier [30]. *Agrobacteria* lines were grown as individual culture at 28°C in YEB medium containing antibiotic selection (100 µg/ml Kanamycin) until each culture reached 0.8 OD_600_. Individual cultures were centrifuged at 7,000 g for 10 min and suspended in infiltration media [50 mM MES (pH 5.6), 0.5% glucose, 2 mM NaPO_4_]. Leaves of *Nicotiana tabacum* (var. Samsun NN) were mechanically infused with each Agrobacterium constructs individually as described earlier [31]. Quantitative measurements of GUS activity were performed 3–4 days post inoculation [25], [26].

### Construction of plant expression vectors and transformation of tobacco plants

Parent and chimeric promoter fragments: Fuas, FSuas, Fcp, FScp, F, FS, FuasFScp, and FSuasFcp were gel eluted after *Eco*RI and *Hind*III restriction digestion of pBSK (+) based clones and sub-cloned into *Eco*RI and *Hind*III sites of plant expressing pKYLXGUS vector [24] by replacing the CaMV 35S promoter. The resulting clones were designated as pKFuasGUS, pKFSuasGUS, pKFcpGUS, pKFScpGUS, pKFGUS, pKFSGUS, pKFuasFScpGUS and pKFSuasFcpGUS respectively, and were used for *Agrobacterium tumefaciens* mediated plant transformation [5], [30]. Twelve independent plant lines were generated for each construct and maintained under green house conditions (photoperiod: 16/8 hrs at 220 µmole m^−2^ s^−1^, Temperature: 28°±3°C, Humidity: 70–75%). Kanamycin-resistant plants (T_1_ generation) were used for further analysis. GUS activity in seedlings was measured according to the protocol described earlier [25], [26]. Transgenic seedlings obtained from each construct were subjected to histochemical GUS staining using 1% X-gluc solution.

### Transformation of *Arabidopsis thaliana* plants

*Arabidopsis thaliana* (ecotype Columbia) plants were transformed by pKYLXGUS, pKFuasFScpGUS and pKFSuasFcpGUS promoter constructs following floral dip method [32] as described earlier [5]. Seeds were collected after maturation and dried. After surface sterilization, seeds were suspended in sterile 0.05% agarose and spread on MS selection plate (4.3 g Murashige & Skoog salts, 10 g sucrose, 0.5 g MES, 8 g agar per liter; pH 5.7, Kanamycin 100 mg/l and Cefotaxime 100 mg/l) and allowed to germinate. Only true transformants produced green healthy leaves (non-transformants became dried and bleached).

### Molecular biology techniques for analyzing transgenic plants

Procedures followed for RNA isolation, northern blot and reverse transcriptase based semi-quantitative PCR analysis of transgenic plants were followed as described earlier in detail [5].

### qRT-PCR (Quantitative Real-Time PCR)

Reactions of qRT- PCR were performed as described earlier [5] with some modifications. The cDNA was synthesized using RNA (*DNase*I treated) isolated from transgenic tobacco plants expressing pKFuasGUS, pKFSuasGUS, pKFcpGUS, pKFScpGUS, pKFGUS, pKFSGUS, pKFuasFScpGUS and pKFSuasFcpGUS promoter construct individually using cDNA synthesis Kit (Fermentas, USA). A standard curve was generated using serially diluted cDNA as described earlier [33]. The qRT- PCR for relative expression analysis was performed using the corresponding cDNA template (1∶15 dilution) and SYBR Premix Ex Taq™ II (Perfect Real Time, Takara Bio Inc., Japan) employing Opticon-2 Real-time PCR machine (MJ Research, Bio-Rad; Model; CFD-3220). Gene specific primers for *GUS* and *GADPH* (Table 1) were used at a concentration of 0.9 µmolar to get 95% efficiency. The absence of genomic DNA contamination was confirmed using minus-reverse-transcriptase controls. The C_t_ value for each reaction was obtained with the help of the software attached with the machine and fold changes in the transcript levels of each construct (considered for qRT-PCR) were presented.

### Histochemical staining and Fluorescent imaging of GUS activity

Whole seedling of transgenic plant (21 days old) and transgenic plant sections expressing the *GUS* gene developed for each constructs were immersed into histochemical GUS staining buffer (100 mM NaPO_4_, 0.5 mM K_3_[Fe(CN)_6_, 0.5 mM K4[Fe(CN)_6_], 10 mM EDTA, 1 mg/ml 5-bromo-4-chloro-3-indolyl-β-D-glucuronide (X-gluc), vacuum infiltrated under pressure for 10 min followed by incubation at 37°C for overnight. Samples were then washed and fixed (in 50% ethanol, 7% acetic acid). The intensity of color development in different tissues was monitored and photographs were taken by using inverted Leica DM LS2 microscope at 10× magnification.

Deciphering of the reporter gene (*GUS*) expression at the cellular/tissue level was carried out by treating the transgenic tissue in 55 µM ImaGene Green™ C12FDGlcU substrate (ImaGene Green™ GUS Gene Expression Kit; Invitrogen, Oregon, USA,) as per kit's instructions and kept under vacuum infiltration for 10 min initially and then incubated at room temperature for 2–3 hrs in the dark. Fluorescence images of the roots of transgenic plants expressing CaMV35S, FuasFScp and FSuasFcp promoter constructs were captured using a CLSM (TCS SP5; Leica, D-68165 Mannheim, Germany). For estimating GUS, the ImaGene Green™ treated stem section and root tissue were excited with 488 diode laser (use of 495 nm UV laser may be more appropriate) and fluorescence emissions were collected between 500 and 515 nm with detector (PMT) gain set at 1150V. GUS localizations at cellular/tissue level were detected by green fluorescent lipophilic fluorescein derivative (5-dodecanoylaminofluorescein) [34].

### Construction of protoplast expression vector with human α-defensin-1 (HNP-1) gene and assay of antimicrobial activity

Human α-defensin-1 (*HNP-1*) gene was PCR-amplified using gene specific primer pair (Table 1) to generate *Xho*I site at 5′ end and *Sac*I at 3′end using HNP-1 clone DNA as a template and PCR conditions: denaturation (94°C for 1 min), annealing (57°C for 45 sec) and extension (72°C for 30 sec) for 35 cycles. The amplified product was gel-purified and digested with *Xho*I and *SacI*, cloned into the corresponding sites of vector pBSK+ to form pBSHNP-1. The defensin gene (*HNP-1*) was isolated as *Xho*I and *Sac*I fragment and cloned into corresponding sites of pUCPMAGUS, pUPFuasFScpGUS, pUPFSuasFcpGUS by replacing the *GUS* gene. The resulting constructs were designated as pUCPMAHNP-1, pUPFuasFScpHNP-1 and pUPFSuasFcpHNP-1 respectively. The DNA sequence integrity of each clone was verified before further use.

Tobacco protoplasts were electroporated with 10 µg of each of the plasmid: pUCPMAHNP-1, pUPFuasFScpHNP-1 and pUPFSuasFcpHNP-1 individually according to the protocol described earlier [24]. Untransformed protoplast was used as a control. After 20 hrs of incubation total soluble protein was isolated by homogenizing the protoplasts in a buffer containing 50 mM Tris-HCl, 5 mM EDTA and protease inhibitor cocktail (Sigma, USA). The homogenate was centrifuged at 10,000× g for 15 min. Supernatant containing protein was collected in a fresh tube and protein concentration was quantified according to [26]. A 100 µl PBS containing 10 µg of protein extracts from protoplasts transformed with each of the above constructs were coated into a 96 well plate. The concentration of protoplast derived HNP-1was estimated following indirect ELISA protocol [35] using an anti-HNP-1 antibody (Santacruz, USA).

Antimicrobial assay of the recombinant peptide was performed as described [36] with slight modification using two bacterial cultures namely *E. coli* (TB1, non-pathogenic) and *Staphylococcus aureus* (pathogenic). In brief, an aliquot of 1.0 ml PBS containing approximately 10^7^ CFU of bacterial cells of *E. coli* (TB1) and *Staphylococcus aureus* individually were centrifuged and resuspended in Mueller-Hinton broth containing 100 µg of protein extract in a final volume of 1.0 ml. These were incubated at 37°C for 2 hrs. An aliquot of 100 µl from 10^5^ dilutions was spread on LB Agar plate, incubated overnight at 37°C and Colony Forming Units (CFU) were counted.

### Statistical analysis

Statistical analysis of all the data was performed adopting one way ANOVA analysis (using GraphPad Prism version 5.01) and presented as a mean of two or three independent experiments. A *P* value of less than 0.05 was considered significant.

## Results

### Comparison of shuffled promoter activities with F, FS and CaMV35S promoter

**Figure 2:**
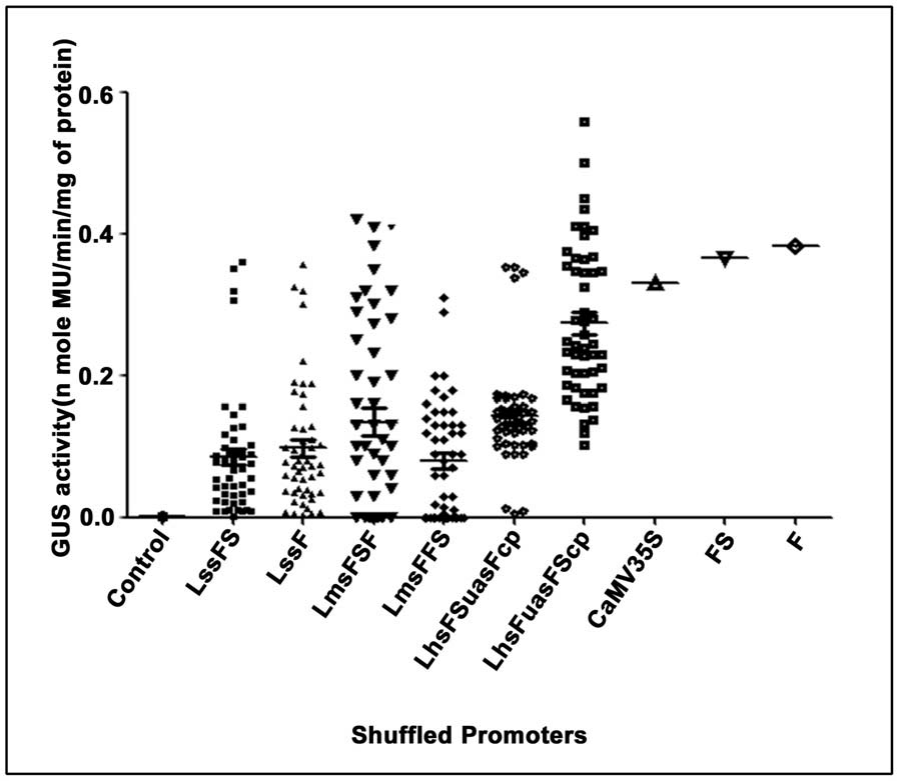
Expression analysis of shuffled promoters in tobacco protoplast transient assay. Fifty shuffled-promoters randomly selected from each of the six libraries (LssF, LssFS, LmsFSF, LmsFFS, LhsFuasFScp and LhsFSuasFcp) were fused with GUS reporter gene. These shuffled promoter-GUS constructs were evaluated along with promoter-GUS constructs of CaMV35S and parent (F and FS) promoters in tobacco protoplast transient assay as described in “Materials and Methods”. The average GUS activity (n mole MU/min/mg protein) of three replicates was presented in the histogram.

**Figure 3:**
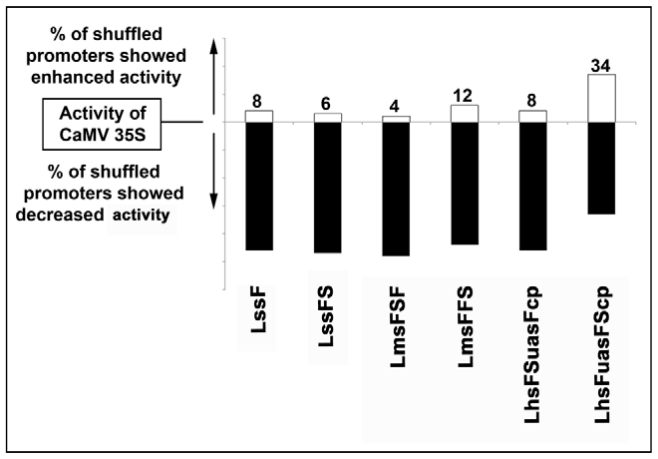
Screening result of shuffled-promoter libraries. The percentage (%) of shuffled promoter clones from six libraries (LssF, LssFS, LmsFSF, LmsFFS, LhsFuasFScp and LhsFSuasFcp) showing enhanced or decreased promoter activity compared to the activity of the CaMV35S promoter was depicted in the histogram.

A schematic flow chart illustrating the construction (Figure 1b) of six shuffled promoter libraries was described in “Materials and Methods” section. A total number of 300 pUCPMAGUS based shuffled promoter clones (50 shuffled promoter clones each from six different shuffled-promoter libraries), and clones with F, FS and CaMV35S promoters fused to the *GUS* reporter gene were evaluated in tobacco (*Nicotiana tabacum* cv. Xanthi Brad) protoplast system. Transformed protoplast with vector (pUCPMA) alone was used as a control. The average GUS activities (obtained from two independent assays) of these shuffled promoter clones along with F, FS and CaMV35S promoters were presented in Figure 2. Most of the shuffled promoters showed reduced activities compared to F, FS and CaMV35S promoters. The result revealed that only 8%, 6%, 12%, 4%, 8% and 34% of shuffled promoters from libraries LssF, LssFS, LmsFFS, LmsFSF, LhsFSuasFcp and LhsFuasFScp, respectively, showed enhanced activity than the CaMV35S promoter respectively (Figure 3).

Comparison of the activities of 300 shuffled promoter clones from six different shuffled libraries with that of the CaMV35S promoter in a transient system, revealed that only 8.66% (26 out of 300) shuffled promoters showed enhanced activity, while only 4.33% (13 out of 300) and 3.6% (11 out of 300) shuffled promoters exhibited enhanced activities compared to FS and F promoters, respectively.

### *In silico* structural analysis of promoter sequences

**Figure 4:**
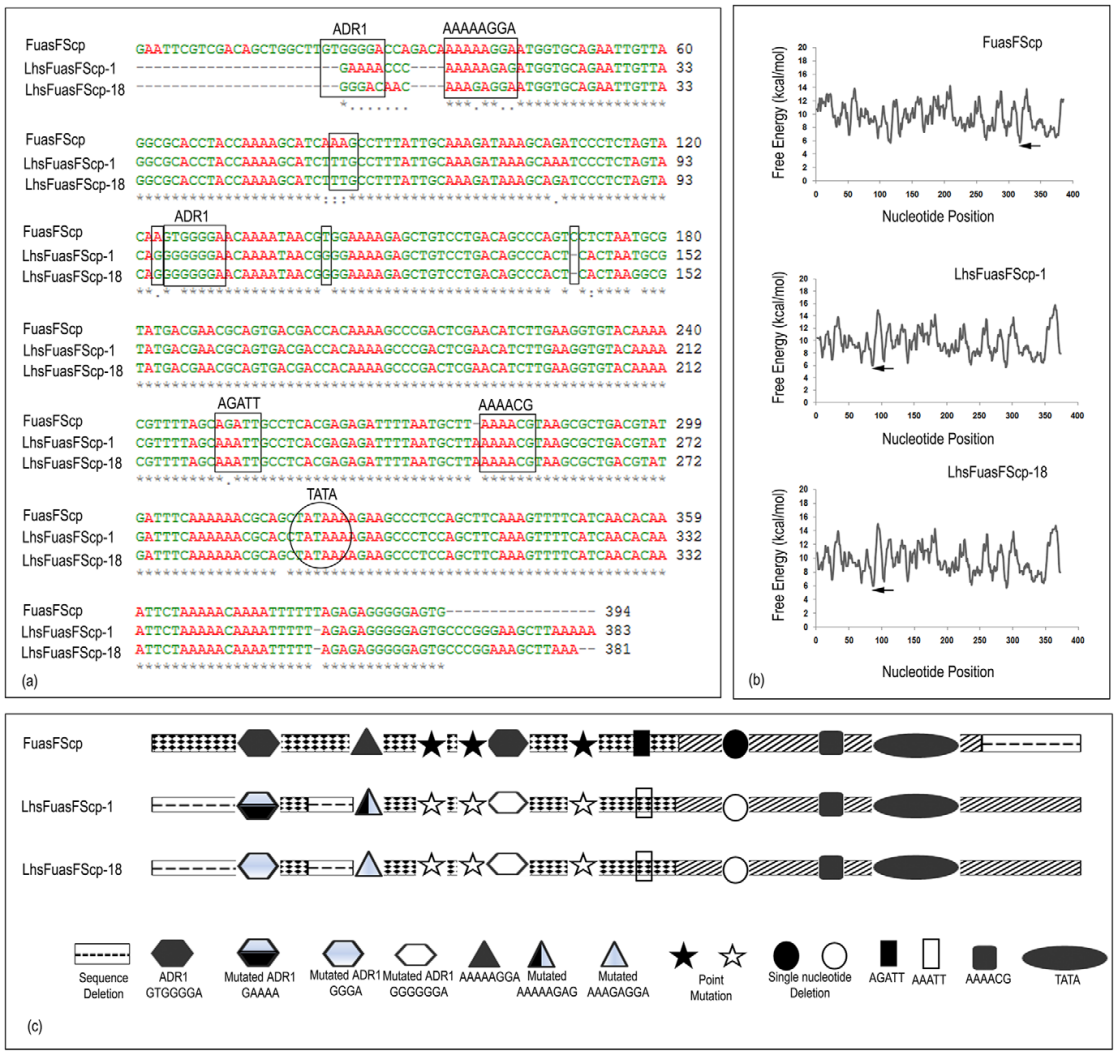
Map of the mutation observed in two shuffled promoters: LhsFuasFScp-1 and LhsFuasFScp-18. (a) Two shuffled promoters LhsFuasFScp-1 and LhsFuasFScp-18 were aligned with the hybrid FuasFScp using ClustalW2 tool (www.ebi.ac.uk/Tools/nsa/clustalw). A square box marks mutation or deletion of important *cis*-elements. The oval shaped box marks TATA elements in these promoter sequences. (b) Free energy profile (helical stability) of each nucleotide present in FuasFScp, LhsFuasFScp-1 and LhsFuasFScp-18 promoter sequences was shown. (c) Diagrammatic representation of location of different *cis*-elements in hybrid promoter FuasFScp. Also point mutations, insertions, deletions in case of two shuffled promoters LhsFuasFScp-1 and LhsFuasFScp-18 were shown.

**Figure 5:**
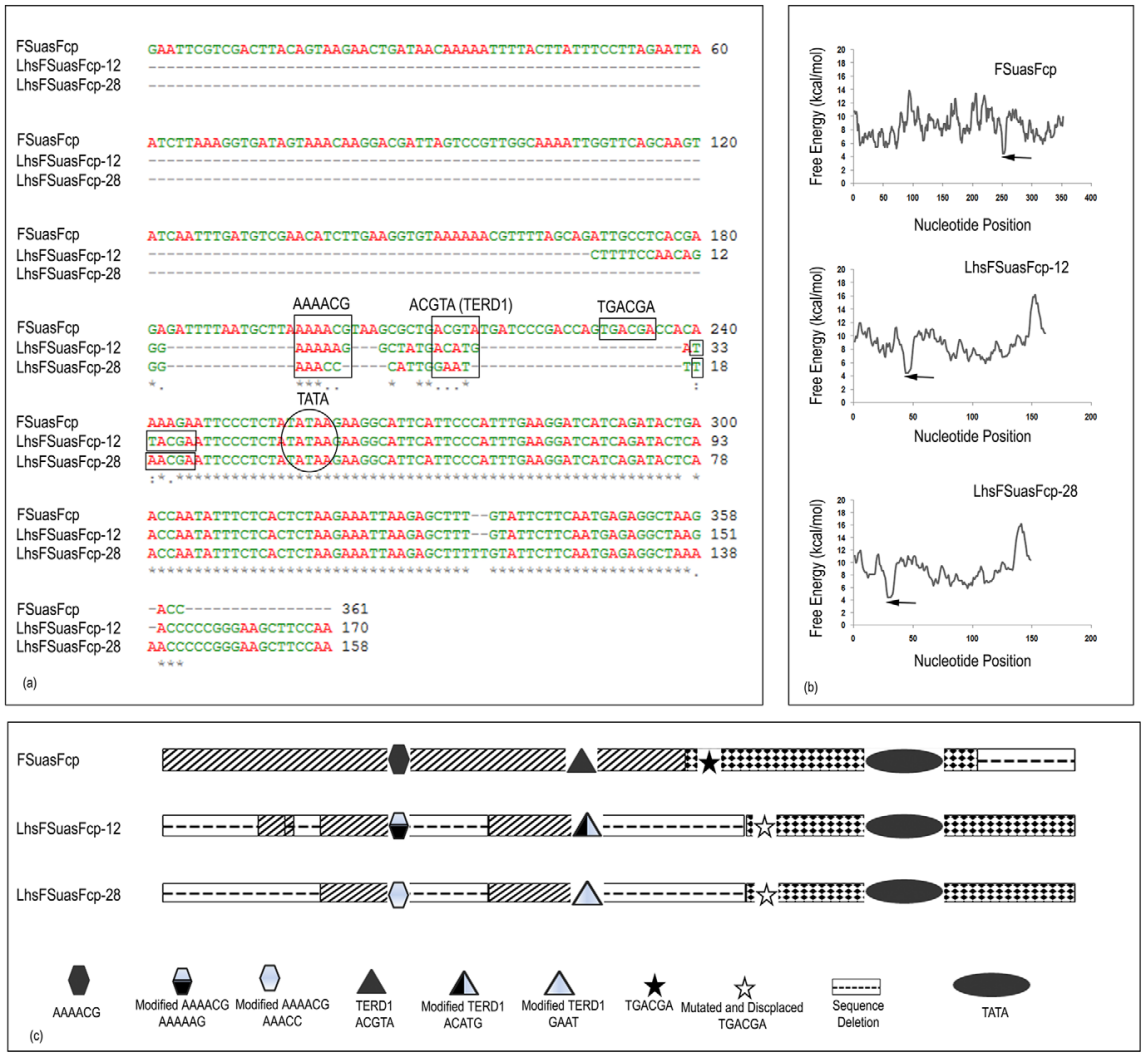
Map of the mutation occurred in two shuffled promoters: LhsFSuasFcp-12 and LhsFSuasFcp-28. (a) The DNA sequence of two shuffled promoters LhsFSuasFcp-12 and LhsFSuasFcp-28 were aligned with the hybrid promoter FSuasFcp using ClustalW2 tool (www.ebi.ac.uk/Tools/nsa/clustalw). A square box marks mutation or deletion of important *cis*-elements. The oval shaped box marks TATA elements in these promoter sequences. (b) Free energy (helical stability) of each nucleotide present in FSuasFcp, LhsFSuasFcp-12 and Lhs-FSuasFcp-28 promoter sequences were shown. (c) Diagrammatic representation of location of different *cis*-elements in hybrid promoter FSuasFcp. Also point mutations, insertions, deletions in case of two shuffled promoters LhsFSuasFcp-12 and LhsFSuasFcp-28 were shown.

The native/natural and rearranged nucleotide sequence between hybrid and hybrid shuffled promoters were compared using ClustalW software (www.ebi.ac.uk/Tools/nsa/clustalw90) [27] to identify the position of different random mutations or deletions in shuffled promoter sequence. We compared two shuffled promoter sequences (LhsFuasFScp-1 and LhsFuasFScp-18) from hybrid shuffled promoter library LhsFuasFScp with the hybrid promoter FuasFScp sequence. We observed several mutations and even deletions of some important *cis*-elements like GTGGGGA (ADR1) in the shuffled promoter (Figure 4a). Point mutations were also observed in the shuffled promoter sequences hsFuasFScp-1 and hsFuasFScp-18 where A to G, T to G and C to A transitions had occurred during shuffling (Figure 4a). Similarly, when we compared two shuffled promoter sequences (LhsFSuasFcp-12 and LhsFSuasFcp-28) from hybrid shuffled promoter library LhsFSuasSFcp with the hybrid promoter FSuasFcp, we observed a deletion of a stretch of sequence containing a number of important specific *cis*-elements like ACGTA-TERD1 among LhsFSuasFcp-12 and LhsFSuasFcp-28 promoter sequences (Figure 5a). Schematic representations of the observations (altered cis-element sequences) were presented in Figures 4c and 5c.

Distortion/melting of the DNA double helix (such as separation of strands and bending of DNA), is necessary for binding of RNA polymerase and other responsible transcription factors at the vicinity of promoter site to form pre-initiation complex [37] Such interaction is a function of enthalpy and entropy (free energy) of the DNA molecule. The free energy of DNA melting is a dinucleotide sequence-dependent (secondary structure event) that is associated with hydrogen bonding energy between AT and GC and base stacking energy. Hence it generates a unique heat map (Free energy pofile) that illustrates the characteristic feature of DNA molecule. Considering all the above factors, a close look into the free energy profile [obtained using the web-thermodyn (http://www.gsa.buffalo.edu/dna/dk/WEBTHERMODYN/) software] of FuasFScp, LhsFuasFScp-1 and LhsFuasFScP-18 promoter sequence; indicated that the free energy profile of the shuffled promoters were significantly different from that of the hybrid promoter (Figure 4b). Similar observations were made with FSuasFcp, LhsFSuasFcp-12 and LhsFSuasFcp-28 promoter sequences also (Figure 5b). These differences could be due to altered state of enthalpy and/or entropy among shuffled and hybrid promoter sequences and finally this may be the cause for reduced activities of LhsFuasFScp-1, LhsFuasFScp-18, LhsFSuasFcp-12 and LhsFSuasFcp-28 promoter constructs.

### Analysis of promoter activities for shuffled, F, FS, FuasFScp and FSuasFcp in whole plant

**Figure 6:**
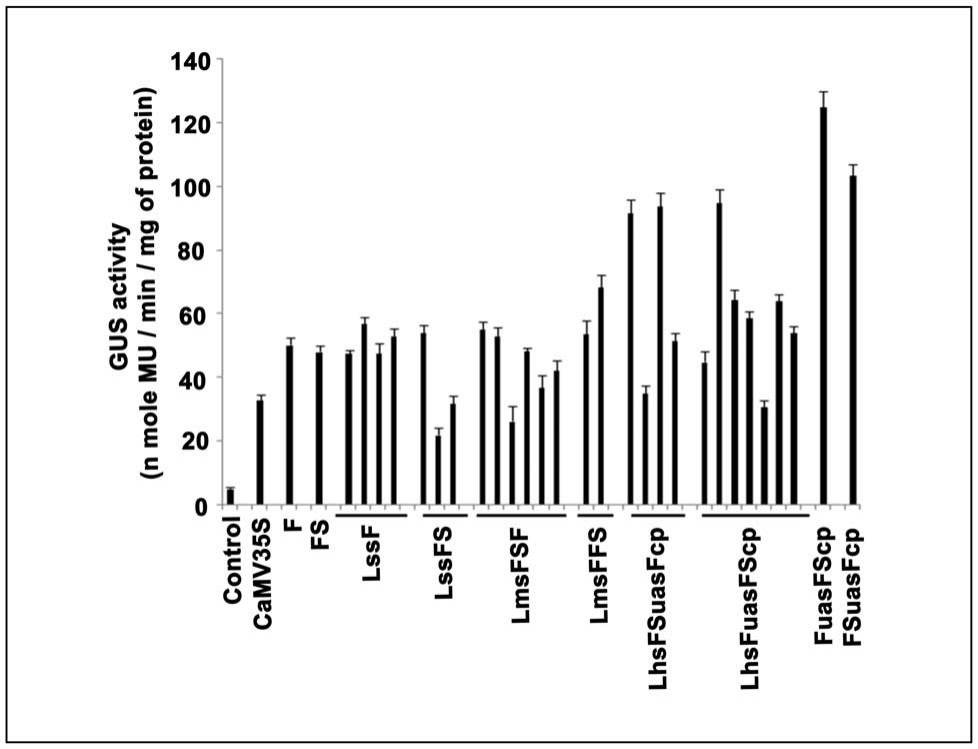
Comparative expression analysis (in Agro-infiltration assay) of selected shuffled promoters screened in protoplast transient assay. In histogram shown, twenty six shuffled promoters giving good activity in transient tobacco protoplast assay selected from six libraries: 4 from LssF, 3 from LssFS, 2 from LmsFFS, 6 from LmsFSF, 4 from LhsFSuasFScp and 7 from LhsFuasFScp; were taken for further comparative expression analysis along with the CaMV35S promoter (35S), parent promoters (F and FS) and hybrid promoters (FuasFScp and FSuasFcp) in Agro-infiltration experiment using whole tobacco plants (*Nicotiana tabacum* samsun NN) as described in “Materials and Methods”. The average GUS activity (n mole MU/min/mg protein ± SD) of three replicates of each construct was presented in the histogram. Error bar shows the 95% confidence intervals of the mean. Statistical (one-way analysis of variance, ANOVA) analysis showed an extremely significant *P* value of <0.001. Empty vector with no GUS gene was treated as ‘Control’.

Shuffled promoters that showed enhanced activities compared to the CaMV35S promoter were considered for further assay. The activities of 26 such promoter clones were compared with that obtained from F, FS, FuasFScp and FSuasFcp promoters using transient agro-infiltration assay in whole tobacco plant as described in “Materials and Methods” section. The activities of each shuffled, parent and hybrid promoter were determined as described earlier. The mean values of three independent experiments along with their respective standard deviations (SD) were presented in Figure 6. The transcriptional activity of the FuasFScp hybrid promoter was found to be higher than the parent promoters (F and FS) or the shuffled promoters studied. We further observed that 2–4% promoter clones in LmsFSF and LhsFuasFScp promoter libraries displayed enhanced activity compared to parent promoters in transient *in vivo* plant assays (Figure 6). The FSuasFcp promoter showed 2.15, 2.05 and 3.13 times stronger activities compared to the parent promoter F, FS and CaMV35S respectively (Figure 6). While the second hybrid promoter, FuasFScp, showed 2.48, 2.59 and 3.79 times stronger activities than F, FS and CaMV35S promoters respectively.

As the activities of shuffled promoter clones were found to be less than that of the hybrid promoters FuasFScp and FSuasFcp; we compared their activities with parent (F and FS) and CaMV35S promoter in the transient protoplast assay using two reporter genes (*GUS* and *GFP*) and evaluated their efficacies in the two independent transgenic plant systems; viz., Tobacco and *Arabidopsis*.

### Comparative expression analysis of F, FS, Fuas, FSuas, Fcp, FScp, CaMV35S, FuasFScp and FSuasFcp promoters fused to *GUS* and *GFP* in tobacco protoplasts

**Figure 7:**
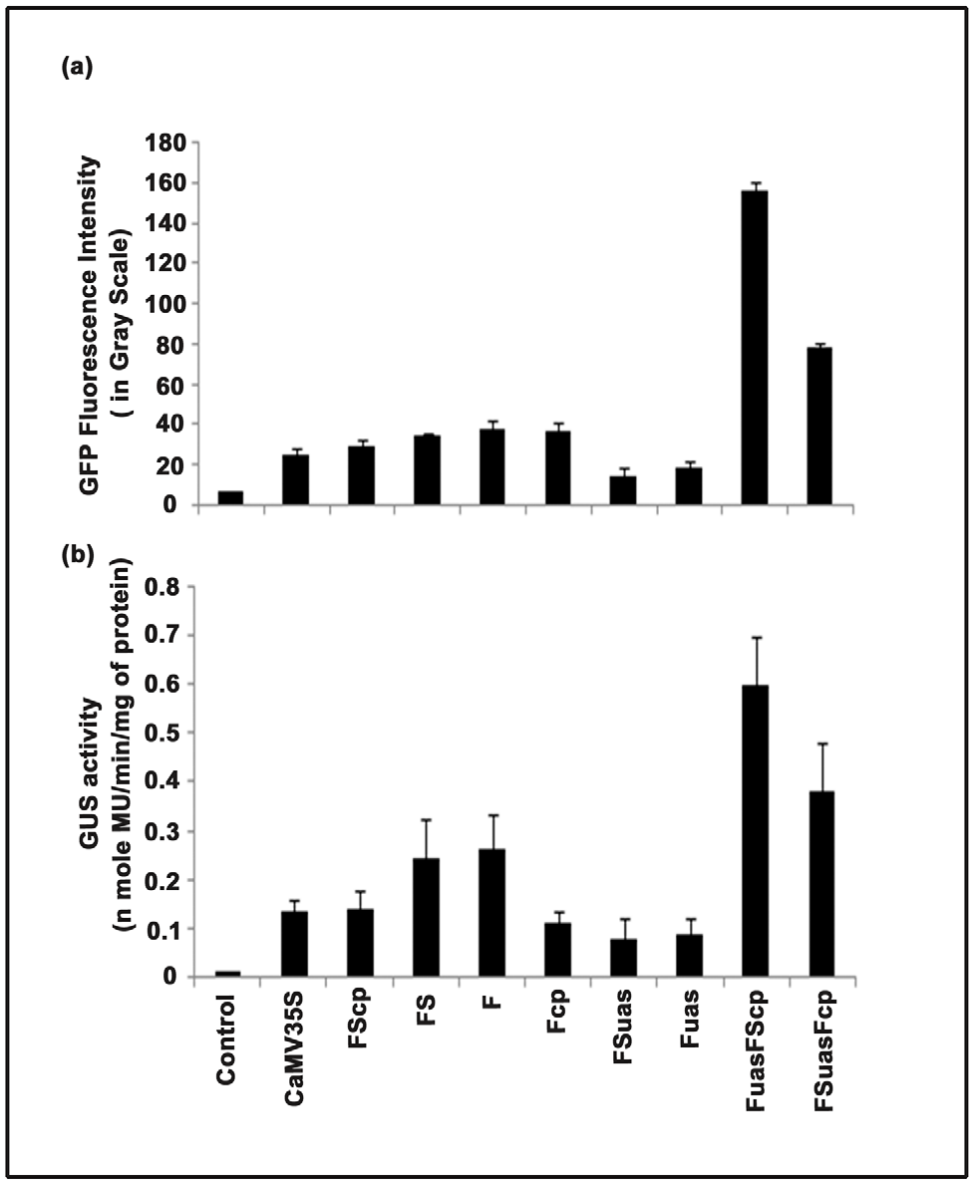
Comparative expression analysis of promoters and promoter fragments fused with reporter genes (GFP and GUS) using CLSM in tobacco transient protoplast assay. (a) GFP constructs of CaMV35S promoter, parent promoters (F and FS), hybrid promoters (FuasFScp and FSuasFcp), promoter fragments (FScp, Fcp, FSuas and Fuas) were created as described in “Materials and Methods”. The GFP fluorescence intensity (in gray scale unit) was measured in protoplast transient assay using CLSM as described in “Materials and Methods”. The average GFP intensity ± SD of two replicates of each construct was presented in the histogram. Error bar shows the 95% confidence intervals of the mean. Statistical (one-way analysis of variance, ANOVA) analysis showed an extremely significant *P* value of <0.01. Empty vector ‘Control’ with no GFP gene was shown. (b) GUS constructs of CaMV35S promoter, parent promoters (F and FS), hybrid promoters (FuasFScp and FSuasFcp), and promoter fragments (FScp, Fcp, FSuas and Fuas) were generated as described in“Materials and Methods”. The GUS activity (n mole MU/min/mg protein) was measured in protoplast transient assay using CLSM as described in “Materials and Methods”. The average GUS activity ± SD of two replicates of each construct was presented in the histogram. Error bar shows the 95% confidence intervals of the means. Statistical (one-way analysis of variance, ANOVA) analysis showed an extremely significant *P* value of <0.02. Empty vector ‘Control’ with no GUS gene was shown.

CLSM-based analysis of the *GFP* reporter (green fluorescence) in promoter constructs demonstrated that the activity of FuasFScp promoter was 2.01, 4.10, 4.58 and 6.24 times stronger than that of the FSuasFcp, F, FS and CaMV 35S promoters, respectively (Figure 7a). The expression level of the *GUS* reporter gene under the control of these promoters was measured as described earlier [5]. It was observed that the FUASFSCP promoter was 1.54, 2.24, 2.36 and 4.45 times more efficient than FSuasFcp, F, FS and CaMV35S promoters (Figure 7b).

### Analysis of F, FS, Fuas, Fcp, FSuas, FScp, CaMV35S, FuasFScp and FSuasFcp promoters in transgenic plants

**Figure 8:**
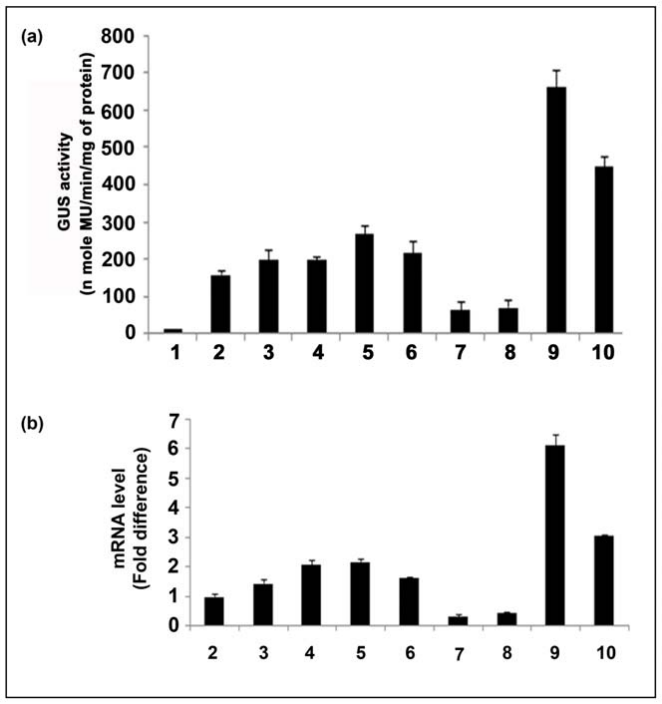
Transgenic analysis of promoter constructs. (**a**) **Comparative stable expression analysis of parent and hybrid promoter-GUS constructs in transgenic tobacco plants.** Promoter activities of parent and hybrid promoters were monitored in 21-days-old tobacco (*Nicotiana tabacum* cv. Samsun NN) seedlings (R1 progeny, 2nd generation, Kan^R^) grown aseptically on an MS-agar medium in presence of kanamycin (300 µg/ml) and 3% sucrose. Soluble protein extracts (5 µg) from whole seedlings were used for the GUS assay. The data presented in the histogram as an average of three independent experiments for each construct with respective standard deviation (SD). The statistical analysis revealed a *P* value of 0.001 implying highly significant. In the histogram, GUS constructs: (1) Untransformed control tissue extract from the wild type *Nicotiana tabacum* cv Samsun NN (2) pKYLXGUS, with CaMV35S promoter, (3) pKFScpGUS, with FScp promoter; (4) pKFSGUS, with FS promoter; (5) pKFGUS, with F promoter; (6) pKFcpGUS, with Fcp promoter; (7) pKFSuasGUS, with FSuas promoter; (8) pKFuasGUS, with Fuas promoter; (9) pKFuasFScpGUS, with FuasFScp promoter; (10) pKFSuasFcpGUS, with FSuasFcp promoter; were shown. (**b**) **Comparative expression analysis of transgenic plants expressing GUS constructs of parent and hybrid promoters by qRT-PCR assay.** For each construct, 21-days-old seedlings (R1 progeny, 2nd generation, Kan^R^) from independent transgenic lines were selected. Estimation of relative *GUS* transcript accumulation in transgenic plants developed using GUS constructs driven by CaMV35S, FScp, FS, F, Fcp, FSuas, Fuas, FuasFScp and FSuasFcp promoters was performed by qRT-RCR as described in “Materials and Methods”. The data presented in the histogram were average fold difference of *GUS* transcript ± SD of two independent experiments carried out using cDNA derived from two RNA samples extracted from two different plants expressing individual promoter constructs. In the histogram, each bar represents number of fold increase in transcript level of *GUS* gene in plants compared to CaMV35S (taken as 1.0). Histograms (2) pKYLXGUS; (3) pKFScpGUS; (4) pKFSGUS; (5) pKFGUS; (6) pKFcpGUS; (7) pKFSuasGUS; 8: pKFuasGUS; (9) pKFuasFScpGUS; (10) pKFSuasFcpGUS were shown.

Total proteins isolated from T_1_ seedlings (21 days old) transformed with the following promoter constructs: pKYLX, pKYLXGUS, pKFuasGUS, pKFSuasGUS, pKFcpGUS, pKFScpGUS, pKFGUS, pKFSGUS, pKFuasFScp and pKFSuasFcpGUS individually were used for GUS activity measurements [25]. The results shown in Figure 8a revealed that in transgenic plants, the FuasFScp promoter exhibited 1.56, 2.21, 2.66 and 4.17 times higher activity, compared to that of FSuasFcp, F, FS and CaMV35S promoters, respectively.

The level of accumulation of *GUS* transcripts in transgenic plants expressing F, FS, Fuas, FSuas, Fcp, FScp, FuasFScp and FSuasFcp promoters was determined using qRT- PCR. The fold differences in the *uid*A-mRNA accumulation levels for F, FS, Fuas, FSuas, Fcp, FScp, FuasFScp and FSuasFcp promoter constructs were presented as the mean of three independent experiments with respective standard deviations (assigning the accumulation level of *uid*A transcript by CaMV35S promoter a value of 1.0) in Figure 8b. As evident from the data, the highest level of accumulation of *uid*A transcripts was observed in transgenic plants carrying the FuasFScp promoter followed by the FSuasFcp, F, FS and CaMV35S promoters.

**Figure 9:**
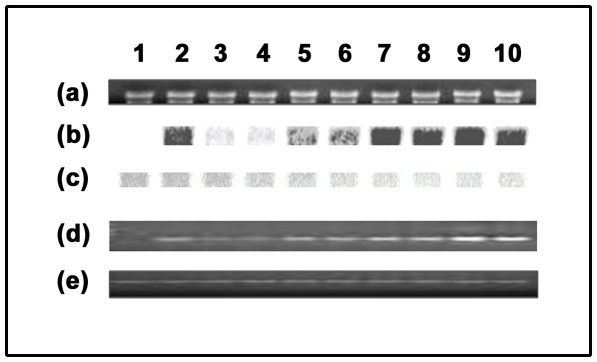
Northern blot and Semi-quantitative PCR analysis of *GUS* transcript using total RNA extracted from transgenic plants. (a) Display of electrophoresis of total RNA obtained from 21-days-old transgenic tobacco seedlings expressing different promoter constructs as discussed in the “Materials and Methods” section. (b) Northern blot analysis of *GUS*-transcript in transgenic tobacco seedling expressing different promoter constructs as described in the “Materials and Methods” section. (c) The same membrane was re-probed with ^32^P-labelled *β-Actin* gene to confirm the equal loading of RNA samples. (d) Electrophoresis of RT-PCR samples of *GUS* transcripts from total RNA (*DNaseI* treated) obtained from transgenic plant expressing different promoter constructs. (e) Electrophoresis of RT- PCR samples of *GAPDH* transcripts from total RNA (*DNaseI* treated) from transgenic plant expressing different promoter constructs. In the figure for the panels a to e, (1) pKYLX (empty vector, transformed plant with no *GUS*); (2) CaMV35S; (3) FSuas; (4) Fuas; (5) Fcp; (6) FScp; (7) F; (8) FS; (9) FuasFScp; (10) FSuasFcp promoter.

The results of Northern analysis for above mentioned promoter constructs were presented in Figure 9b to further confirm the result obtained from qRT-PCR. We observed the strongest signal after northern blot hybridization (GUS transcripts accumulation) for the FuasFScp promoter, followed by the FSuasFcp, F, FS and CaMV35S promoters using MultiGuage ver 2.0 software (data not shown). The result of β-*Actin* used as loading control was shown in Figure 9c.

The result of PCR amplifications on cDNA obtained after reverse transcription of total RNA for *GUS* gene driven by the above cited promoters in transgenic plants were displayed in Figure 9d.

### Histochemical staining

**Figure 10:**
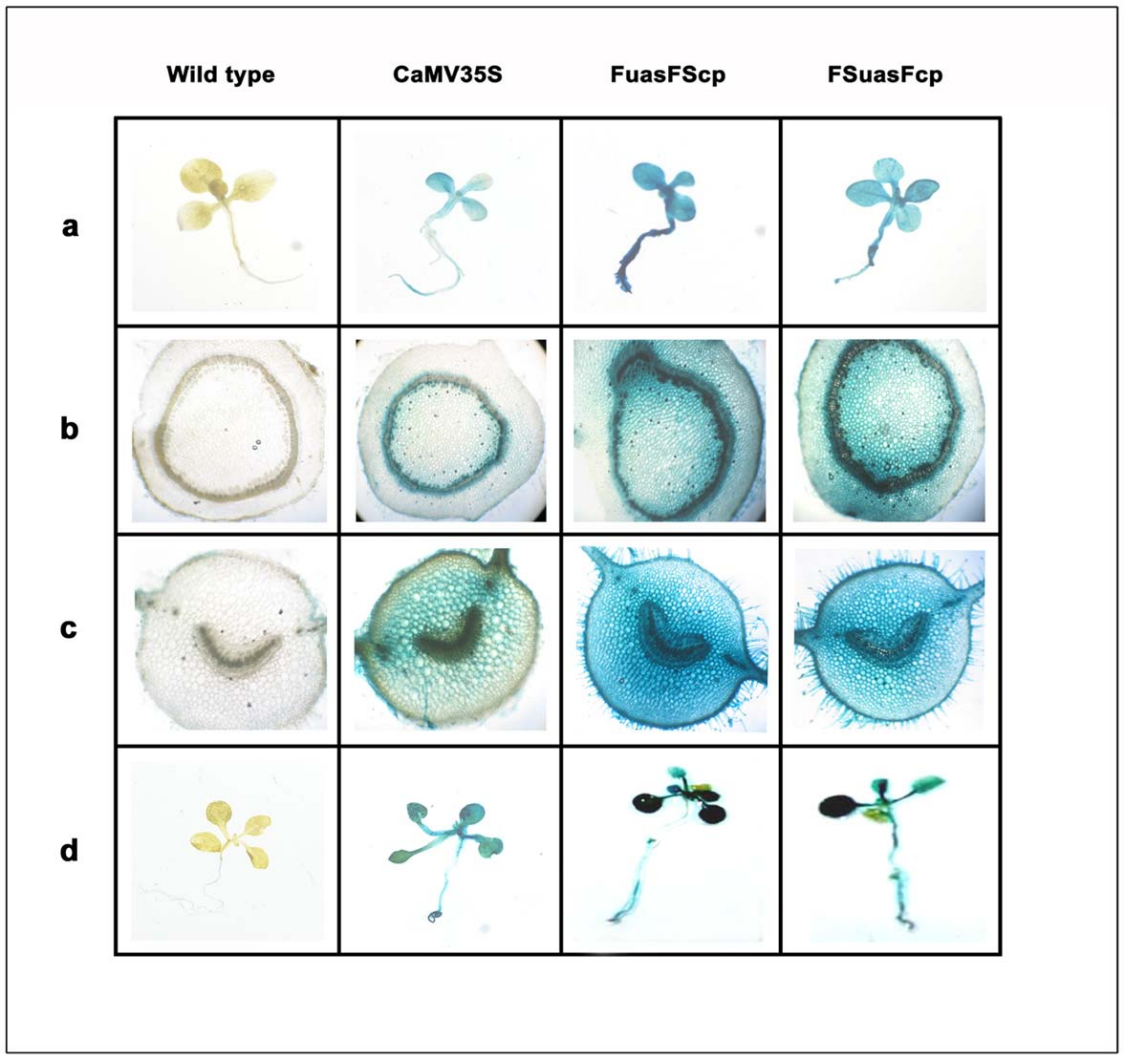
Histochemical localization of GUS activity in transgenic tobacco and Arabidopsis seedlings generated for the respective promoter-*GUS* constructs. (a) Histochemical staining of transgenic tobacco seedlings expressing GUS under the control of respective promoter constructs. Photographs were taken using Leica DM LS2 microscope (at 10× magnification) attached to a CCD camera. (b) Histochemical staining of transgenic tobacco stem cross sections expressing GUS under the control of respective promoter constructs. Photographs were taken using Leica DM LS2 microscope (at 10× magnification) attached to a CCD camera. (c) Histochemical staining of transgenic tobacco leaf petiole cross sections expressing GUS under the control of respective promoter constructs. Photographs were taken using Leica DM LS2 microscope (at 10× maginification) attached to a CCD camera. (d) Histochemical staining of transgenic *Arabidopsis* seedling expressing GUS under the control of respective promoter constructs. Photographs were taken using Leica DM LS2 microscope (at 10× maginification) attached to a CCD camera.

Histochemical staining using X-gluc (5-bromo-4-chloro-3-indolyl-β-D-glucuronide) of transgenic tobacco seedlings (T_1_ generation, 21 days old) generated for the GUS construct with CaMV35S; FuasFScp and FSuasFcp promoter were presented in Figure 10a, while those of stem cross sections of transgenic tobacco plants and of leaf petioles were presented in (Figures 10b and 10c, respectively). Histochemical staining of transgenic *Arabidopsis* seedlings expressing GUS directed by CaMV35S, FuasFScp and FSuasFcp promoters were shown in Figure 10d.

**Figure 11:**
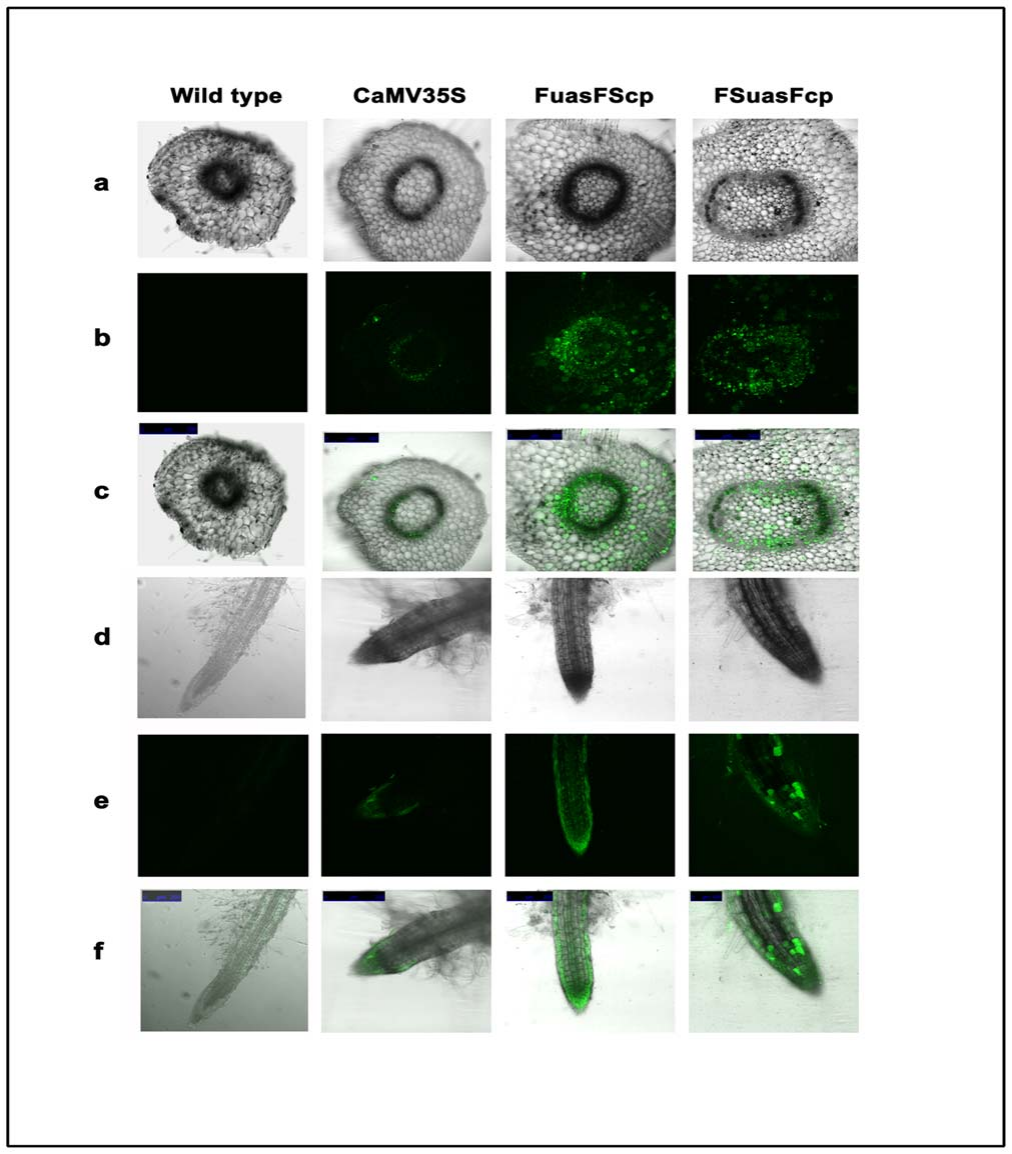
CLSM based analysis of localized GUS expression in transgenic tobacco expressing respective promoter-*GUS* constructs. (a) Bright field confocal images of transverse sections of transgenic tobacco stem expressing GUS under the control respective promoter constructs. (b) Fluorescence images of transverse sections of transgenic tobacco stem expressing GUS under the control of respective promoter constructs. (c) Superimposed (bright field and fluorescent) images of transverse sections of tobacco stem expressing GUS under the control of respective promoter constructs. (d) Bright field confocal images of transgenic tobacco root expressing GUS under the control of respective promoter constructs. (e) Fluorescence images of tobacco root expressing GUS under the control of respective promoter constructs. (f) Superimposed (bright field and fluorescent) images of tobacco root expressing GUS under the control of respective promoter constructs. Images were captured using CLSM as described in “Materials and Methods”. Plant samples used were grown aseptically under tissue culture conditions. All figures under row a, b and c were presented in 500 micrometer (µm) scale while figures under row d, e and f were presented in 250 micrometer (µm) scale except figures obtained from promoter FSuasFcp [presented using 100 micrometer (µm) scale].

Fluorescence images of stem cross section and root tissue of transgenic plants expressing *GUS* gene with CaMV35S, FuasFScp and FSuasFcp promoter treated with ImaGene Green™ C12FDGlcU substrate were captured using a CLSM (TCS SP5; Leica, D-68165 Mannheim, Germany). Data were presented in Figures (11a–f).

### Comparative expression analysis of pUCPMAHNP-1, pUPFuasFScpHNP-1 and pUPFSuasFcpHNP-1 constructs in tobacco protoplasts

The protoplast expression constructs pUCPMAHNP-1; pUPFuasFScpHNP-1 and pUPFSuasFcpHNP-1 were generated as described in “Materials and Methods”. The HNP-1 peptide gene was expressed in tobacco protoplast under the control of CaMV35S, FuasFScp and FSuasFcp promoters. The concentration of HNP-1 peptide in protoplast extracts was measured by ELISA using anti-HNP antibody as described in “Materials and Methods”. In protoplast expression experiment, CaMV35S, FuasFScp and FSuasFcp promoters derived HNP-1 concentrations (wt/wt) were estimated to be 8.2 µg, 29.1 µg and 10.2 µg per mg of total soluble crude protein in crude protoplast extracts, respectively. In this context, the level of expression of HNP-1 under CaMV35S, FuasFScp and FSuasFcp promoter was about 0.8%, 2.9% and 10.2% of total soluble protein, respectively.

**Figure 12:**
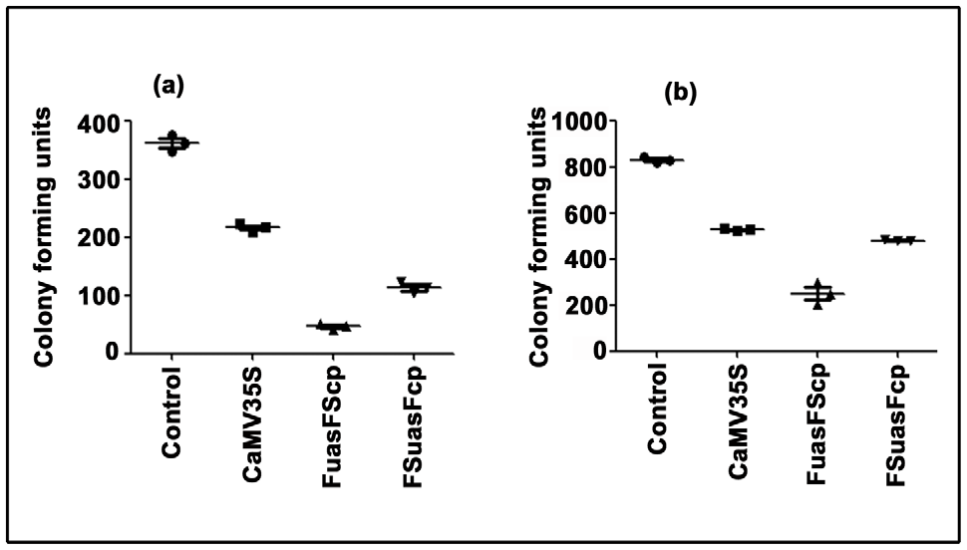
Comparative expression analysis of hybrid promoters (FuasFScp and FSuasFcp), and CaMV 35S promoter fused with human alpha defensin-1 (HNP-1) gene in tobacco protoplasts. The human alpha defensin-1 (HNP-1) gene was expressed in protoplasts under the control of CaMV 35S, FuasFScp and FSuasFcp promoters as described in “Materials and Methods”. Antibacterial assay of human alpha defensin-1 (HNP-1) protein extracted from tobacco protoplasts was performed using (a) *Escherichia coli* cell and (b) *Staphylococcus aureus* cell as described in “Materials and Methods” section. The data were presented as a mean of two independent experiments with respective SD.

The antibacterial activity of HNP-1 peptide in tobacco protoplast extracts was assayed using *E. coli* cells (TB1) and *Staphylococcus aureus* separately as described in “Materials and Methods”s. The antimicrobial activity assay data were presented in Figure 12a and 12b respectively. The antibacterial activity of FuasFScp promoter-driven HNP-1 showed 4.6 and 2.44 times stronger antibacterial activity assayed with *E. coli* (TB1) cells; and 2.11 and 1.92 times stronger activities assayed with *Staphylococcus aureus* compared to FSuasSFcp and CaMV35S promoters, respectively.

## Discussion

With a view to develop efficient plant promoters, we generated chimeric promoters through DNA shuffling, hybridization and hybridization-shuffling techniques using two well characterized (heterologous) promoters, namely, *Figwort mosaic virus* full-length (F, −249 to +64, from TSS) [22] and sub-genomic-transcript (FS, −270 to +31, from TSS) [23] promoters.

The majority of the shuffled promoters showed reduced activities when compared to the parent (F and FS), hybrid (FuasFScp and FSuasFcp) and CaMV35S promoters. Only 8%, 4% and 3% shuffled promoter showed increased activities compared to the CaMV35S, FS and F promoters respectively. This observation thus partly supports the earlier findings that chimeric shuffled promoters developed from *Banana Streak Virus* [38] and *Cauliflower Mosaic Virus* [39] using DNA shuffling approach showed reduced activities as seen in our study. We obtained a few shuffled promoters with higher activities compared to F, FS and CaMV35S promoter.

The activities of hybrid promoters were found to be higher than those of F, FS and CaMV35S promoters. The enhanced activities of the hybrid promoters may arise due to the free energy content of the promoter DNA sequence. The free energy usually depends on the near neighbor-hood interaction and the GC content of the sequence and the free energy at regulatory regions (TATA box) plays a vital role in localized unwinding of the DNA double helix for RNA polymerase and other protein factors to bind thereby facilitating transcription process [40]. We observed a significant difference in the free-energy profiles between the (hybrid) and hybrid-shuffled promoter sequences, the free energy of shuffled promoter sequences being less compared to that of hybrid promoters indicating their higher stability (Table 2). Probably this might be one of the reasons for reduced activity of the shuffled promoters.

**Table 2:** Free energy Profile and GC content of shuffled and hybrid promoters.

S.N	Promoter	GC%	Free Energy
1	FuasFScp	42.9	422.76
2	LhsFuasFScp-1	42.0	413.53
3	LhsFuasFScp-18	43.0	412.94
4	FSuasFcp	36.0	344.40
5	LhsFSuasFcp-12	38.8	168.77
6	LhsFSuasFcp-28	37.3	152.46

As all the shuffled promoters from six shuffled libraries showed reduced activity than hybrid promoters FuasFScp and FSuasFcp, we continued our further investigation with hybrid promoters to test their overall efficacy and potential to be used in plant genetic engineering. Hybrid promoter showed enhanced activity compared to parent promoters (F and FS) and CaMV35S promoters in both transient and transgenic plants (Tobacco and *Arabidopsis*). These observations were further validated through *uidA* transcript assay using Northern blot and qRT-PCR analysis. Histochemical staining experiments also supported above observations. Spatial expression pattern of these hybrid promoters indicate that activities of hybrid promoters were distributed differentially among different cell/tissue types of plant root, leaf and stem. Analyzing the ImaGene Green™ based fluorescent images of plant root and stem using the LAS-AF software attached to CLSM, it was observed that in Gray-Scale unit the intensities of green coloration in root tissue were 153.63, 105.51 and 44.41 for FuasFScp, FSuasFcp and CaMV35S respectively (after adjusting the background control of 30.75 unit in Gray-Scale. Interestingly, we observed the activities of these promoters were localized mostly in the meristematic region of root tip compared to the CaMV35S promoter. Our results on antimicrobial assay of protoplast-derived human alpha defensin-1 (HNP-1) [41], [42] clearly reflected the potential of these hybrid promoters to be used as candidate promoters for plant transgenic research in molecular farming/plant made product (PMP) applications.

All the six shuffled libraries mentioned above contain promoters with a broad spectrum of (varying) activity; out of which promoter with both reduced and enhanced activities could be used in plant biotechnology applications like engineering a metabolic pathway and plant molecular farming. As the promoters with both high and low activity are needed depending upon the situation, each of the promoters we generated under these six libraries, provides a rich resource of promoter/s for plant genetic engineering. Our results clearly suggest that the hybrid promoters, viz., FuasFScp and FSuasFcp with enhanced activity and near constitutive in nature in combination with shuffled promoters could be potentially useful in both public sector and academia.

It is well established in literature that others have successfully used *DNaseI* shuffling or molecular evolution for enhancing the activity of gene (protein) by several hundred folds. However in our study we observed the percentage of promoter clones showing enhanced activity is minimal. Such discrepancy could arise due to the fact that in our case only one round of shuffling was performed. Probably several rounds of shuffling and more intensive screening are required to develop a promoter with ‘super- activity’.

### Conclusion

The efficiency with which a promoter functions is largely dependent on the presence of an intact core structure containing two functional cross-talking/interacting domains; viz., upstream activation sequence (uas) and downstream core promoter sequence. Many efforts are thus needed to develop a useful promoter through shuffling and we assume that multiple rounds of shuffling and more intense screening would help in generating the desired promoter. Any deviation from this core structure may lead to the loss of desired function (promoter activity). Shuffled promoter, developed through genetic rearrangement in an uncontrolled manner, fully loaded with mutation (insertion/deletion) or with a deformed structure may not always result in a useful promoter. A promoter, as hybrid or synthetic, usually developed through precise/specific controlled genetic manipulation and retaining their core structure is more likely to lead to be a useful promoter. The hybrid promoters, FuasFScp and FSuasFcp developed in this study could be useful in engineering gene-constructs suitable for ectopic expression of various genes in transgenic plants and could thus prove to be potential candidate promoters in plant genetic engineering and translational research.
